# Neonatal Platelets: Lower G_12/13_ Expression Contributes to Reduced Secretion of Dense Granules

**DOI:** 10.3390/cells11162563

**Published:** 2022-08-17

**Authors:** Axel Schlagenhauf, Sheila Bohler, Mirjam Kunze, Tanja Strini, Harald Haidl, Miriam Erlacher, Barbara Zieger

**Affiliations:** 1 Division of General Pediatrics, Department of Pediatrics and Adolescent Medicine, Medical University of Graz, 8036 Graz, Austria; 2 Division of Pediatric Hematology and Oncology, Department of Pediatrics and Adolescent Medicine, Faculty of Medicine, Medical Center-University of Freiburg, 79098 Freiburg im Breisgau, Germany; 3 Department of Obstetrics and Gynecology, Faculty of Medicine, University of Freiburg, 79110 Freiburg im Breisgau, Germany

**Keywords:** neonatal platelets, platelet secretion, dense granules, GPCRs, RhoA

## Abstract

Despite fully functional primary hemostasis, platelets of healthy neonates exhibit hypoaggregability and secretion defects, which may be adaptations to specific requirements in this developmental stage. The etiologies for reduced signal transduction vary with the type of agonist. The discovered peculiarities are lower receptor densities, reduced calcium mobilization, and functional impairments of G proteins. Reduced secretion of dense granules has been attributed to lower numbers of granules. Signaling studies with adult platelets have shown a regulating effect of the G_12/13_ signaling pathway on dense granule secretion via RhoA. We comparatively analyzed secretion profiles using flow cytometry and expression levels of G_q_, G_i_, and G_12/13_ using Western blot analysis in platelets from cord blood and adults. Furthermore, we evaluated Rho activation after in vitro platelet stimulation with thrombin using a pulldown assay. We observed a markedly reduced expression of the dense granule marker CD63 on neonatal platelets after thrombin stimulation. G_α12/13_ expression was significantly decreased in neonatal platelets and correlated with lower Rho activation after thrombin stimulation. We conclude that lower expression of G_12/13_ in neonatal platelets results in attenuated activation of Rho and may contribute to reduced secretion of dense granules after exposure to thrombin.

## 1. Introduction

In healthy individuals, coagulation is tightly regulated to maintain the hemostatic balance between bleeding and thrombosis. The developing hemostatic system matures in several phases from fetus to adult, gradually changing and adapting to the requirements of each developmental stage [1,2,3]. Consequently, neonatal hemostasis differs from that of adults and has several peculiarities [4,5]. Despite lower levels of clotting factors, healthy neonates do not exhibit a bleeding tendency, because lower levels of inhibitors keep the neonatal hemostatic system in balance at a lower level [6]. The fragility of this delicate balance correlates with gestational age, making preterm neonates more prone than term neonates to bleeding as well as thrombosis [7,8].

Neonatal primary hemostasis also exhibits several peculiarities. While platelet counts and size in healthy neonates are comparable to those of adult controls [9,10], neonatal platelets show impaired functionalities, such as a lower response to various agonists [11], reduced aggregability [12,13], and less secretion [14]. On the other hand, the bleeding times and PFA-100 closure times of neonates are even shorter than those of adults, which is probably attributable to a higher hematocrit and a larger fraction of high-molecular-weight von Willebrand factor, resulting in improved platelet adhesion [15,16]. A moderated platelet function may be required to maintain the fragile hemostatic balance in neonates. Platelets also play a crucial role in secondary hemostasis; thus, transfusion of adult platelets into thrombocytopenic neonates can create a developmental mismatch that leads to an excess of prothrombotic surfaces [17].

The nature of neonatal platelet peculiarities has been the subject of several studies because intracranial hemorrhage associated with platelet dysfunction is a widely feared complication in preterm neonates. Most studies use cord blood as a source for neonatal platelets. It is available in ample amounts, and a direct comparison to platelets from neonatal venous blood did not show differences in platelet count, platelet volume, surface glycoproteins, or activation behavior [18].

Early studies of the functional structure of neonatal platelets showed lower surface expression of GPIbα (CD42b) and varying expression of P-selectin and integrin αIIbβ3 depending on gestational age [19,20,21,22]. Neonatal platelets exhibit reduced granule secretion upon stimulation, resulting in less shedding of bioactive molecules into circulation [3,14]. We showed that inorganic polyphosphate (PolyP), a prothrombotic substance accumulated in platelet dense granules, is shed to a lesser extent by neonatal platelets than adult platelets. Plasmatic coagulation in neonates appears to be more sensitive than that of adults to prothrombotic stimuli of platelet-derived PolyP, emphasizing the notion that platelet function is tailored to developmental requirements [23,24].

Studies focusing on neonatal platelet signaling revealed that platelets of healthy neonates exhibit alterations in several signaling pathways, including reduced collagen and thromboxane A2 (TXA_2_) signal transduction to phospholipases A2 and Cβ, leading to impaired mobilization of intracellular calcium [25,26]. Response to epinephrin is reduced due to lower surface density of α2-adrenergic receptors on neonatal platelets. Equivalently, our group showed that the reduced response of neonatal platelets to thrombin is caused by lower levels of protease-activated receptors (PAR1 and PAR4) [27].

These receptors are coupled to various types of G-proteins (GPCRs) to mediate signaling upon thrombin stimulation. The known G-protein subtypes coupled with PARs in platelets are G_q_, G_i_, and G_12/13_. G_q_ signaling is dependent on the activation of phospholipase Cβ (PLCβ) to induce integrin activation, shape change, and amplification via synthesis of TXA_2_ [28]. G_i_ signaling, particularly via G_i2_, has been shown to inhibit adenylyl cyclase, thus blocking the synthesis of cyclic adenosine monophosphate (cAMP) [29]. Since cAMP is a general suppressor of platelet signaling, switching off its synthesis via G_i_ signaling is a way for platelet agonists to trigger activation pathways. G_12_ and G_13_ are highly homologous, and both mediate signaling via activation of the small GTPase RhoA, which is required for cytoskeletal rearrangement, leading to platelet shape change [30,31]. Studies on PAR signaling showed that thrombin-mediated G_q_ signaling is not sufficient for platelet secretion and that the G_12/13_ signaling pathway, through RhoA activation, regulates dense granule release [32].

Israels et al. observed that stimulation of neonatal platelets with the TXA_2_ mimetic U46619 led to reduced activation of PLCβ compared to adult platelets. Subsequently, they analyzed the expression of G_q_ in neonatal and adult platelets but found comparable levels. Finally, they observed a reduced GTPase activity of the G_αq_-subunit coupled to the TXA_2_ receptor of neonatal platelets, explaining the hyporeactivity of neonatal platelets to TXA_2_ [26].

Since we observed an underexpression of thrombin receptors on neonatal platelets [27], we hypothesized that G proteins required for thrombin signaling may be also differentially expressed affecting platelet neonatal platelet secretion. Hence, we conducted a study that compared the expression of G proteins in neonatal and adult platelets in combination with platelet secretion analyses.

## 2. Materials and Methods

### 2.1. Participants and Preanalytics

This study was carried out in accordance with the recommendations of the Ethics Committee of the Albert-Ludwigs University Freiburg (protocol code 353/16). All adult participants gave written informed consent in accordance with the Declaration of Helsinki. All mothers gave their informed consent in writing to obtain cord blood. The protocol was approved by the Freiburg Institutional Review Board.

Cord blood was obtained from full-term infants (N = 11) with uncomplicated delivery after 39–42 weeks of gestation immediately after accouchement, anticoagulated with trisodium citrate (0.106 M). Venous blood was obtained from informed healthy adult volunteers (N = 11) who had not taken any medication affecting platelet function for at least 2 weeks before the study. With loose application of a tourniquet, venous blood was drawn from the antecubital vein and anticoagulated with trisodium citrate (0.106 M). Samples from both cohorts were processed within 1 h after collection to prevent cellular activation. Platelet-rich plasma (PRP) was prepared from all blood samples by differential centrifugation (200× *g*, 10 min) at room temperature.

### 2.2. Platelet Granule Secretion Assay

Platelet granule secretion was determined by flow cytometric analysis of surface expression markers for α-granules (CD62) and dense granules (CD63) after stimulation with increasing amounts of thrombin. PRP was diluted in autologous plasma to a concentration of 5 *×* 10^7^ platelets/mL. Platelets in PRP were stimulated using increasing concentrations of thrombin (0, 0.05, 0.1, 0.2, 0.5, and 1.0 U/mL) in the presence of 1.25 mM of the peptide Gly-Pro-Arg-Pro (GPRP) to prevent fibrin polymerization [33]. The reaction was stopped after 3 min by fixation with 1% formaldehyde in PBS for 30 min. After fixation, cells were washed in 500 μL PBS and incubated with FITC (fluorescein isothiocyanate)–conjugated anti-CD62 or FITC-conjugated anti-CD63. The expression of surface fluorescence was analyzed with a FACSCalibur flow cytometer (Becton Dickinson, Franklin Lakes, NJ, USA). Analyses were performed with neonatal platelets in pairs with platelets from healthy controls. Data were analyzed as linear arbitrary units and expressed as % of maximum of adult controls.

### 2.3. Western Blot Analysis of G-Protein Expression

PRP was diluted (1:1 *v*/*v*) with citrate buffer (120 mM NaCl, 12.9 mM citrate, 30 mM glucose, pH 6.5) and incubated for 10 min at room temperature. Then, platelets were sedimented by centrifugation (400× *g*, 10 min) and resuspended in HEPES buffer (145 mM NaCl, 5 mM KCl, 1 mM MgCl_2_, 10 mM HEPES, 10 mM glucose, pH 7.4). A subfraction of this platelet suspension was subjected to stimulation with thrombin (0.5 U/mL) in the presence of 2 mM CaCl_2_. Resting or activated platelets were lysed by adding a concentrated lysis buffer containing sodium dodecyl sulfate (SDS) (1:4, *v*/*v*; 4% SDS, 150 mM NaCl, 50 mM Tris, pH 7.4). Platelet lysates were vortexed and stored at −80 °C until further processing.

In total, 10 μg of protein were resolved by sodium dodecyl sulfate-polyacrylamide gel electrophoresis and blotted onto nitrocellulose membranes (Invitrogen, Thermo Fisher Scientific, Waltham, MA, USA), followed by immunodetection with mouse antibodies against G_αq_, G_αi2_, and G_α12/13_ (Santa Cruz Biotechnology, Dallas, TX, USA) and a goat anti-mouse IgG-HRP antibody (Santa Cruz Biotechnology, Dallas, TX, USA). GAPDH served as loading and staining control. Blot bands were detected with a Molecular Imager Chemi DocTM XRST Imaging System (Bio-Rad). Densitometric analysis was performed with Image Lab 6.1 (Bio-Rad Laboratories, Hercules, CA, USA). The densities of G-protein bands were normalized to GAPDH. We focused on the α-subunits of respective G-protein isoforms, since they are known to transduce PLCβ activation, adenylyl cyclase inhibition, and RhoA activation upon receptor binding [34]. We assumed that the expression of an isoforms α-subunit corresponded to the expression level of the whole G-protein.

### 2.4. Rho Activation Assay

Washed platelet suspensions were prepared as described above, and a subfraction was stimulated with thrombin. Rho activation was determined using a commercial active Rho pull-down and detection kit (Thermo Fisher Scientific). The assay is based on the specific pull-down of active Rho bound to GTP via its interaction with the Rhotekin protein-binding domain.

This assay does not distinguish between highly homologous Rho variants, but RhoA is the dominantly expressed isoform in human platelets [31]. Briefly, platelets were pelleted by centrifugation (400× *g*, 10 min) and lysed in binding buffer (25 mM Tris*HCl, 150 mM NaCl, 5 mM MgCl_2_, 1% NP-40, 5% glycerol, pH 7.2) containing Halt protease and phosphatase inhibitor cocktail (Thermo Fisher Scientific). The protein content was determined using BCA-assay (Thermo Fisher Scientific) and 1 mg protein was subjected to active Rho pulldown using GST-fusion protein of the Rhotekin-binding domain (RBD) along with glutathione agarose resin. Active Rho was removed from the resin using β-mercaptoethanol and SDS, and detection was done by Western blot analysis using a rabbit anti-Rho antibody and a goat anti-rabbit IgG-HRP antibody. Detection of blot bands and densitometric analysis were performed as described above. Blot band densities were normalized to the average of adult values.

### 2.5. Statistics

Data are presented as mean ± SD. CD62/CD63 receptor densities on neonatal and adult platelets at increasing thrombin concentrations were analyzed using ANOVA. Corrections for multiple comparisons were made using the Holm-Šídák method (α = 0.05), and multiplicity-adjusted *p*-values were calculated for each comparison. Differences between cord blood or adult samples were analyzed using Student’s *t*-test or the Man–Whitney U-test. Correlations were calculated using Pearson’s correlation coefficient. Graphpad Prism 6.0 (Graphpad software, San Diego, CA, USA) was used for performing calculations and creating figures. The datasets generated and/or analyzed during the current study are available from the corresponding author on request.

## 3. Results

### 3.1. Secretion Markers of Thrombin-Stimulated Neonatal and Adult Platelets

**Figure 1:**
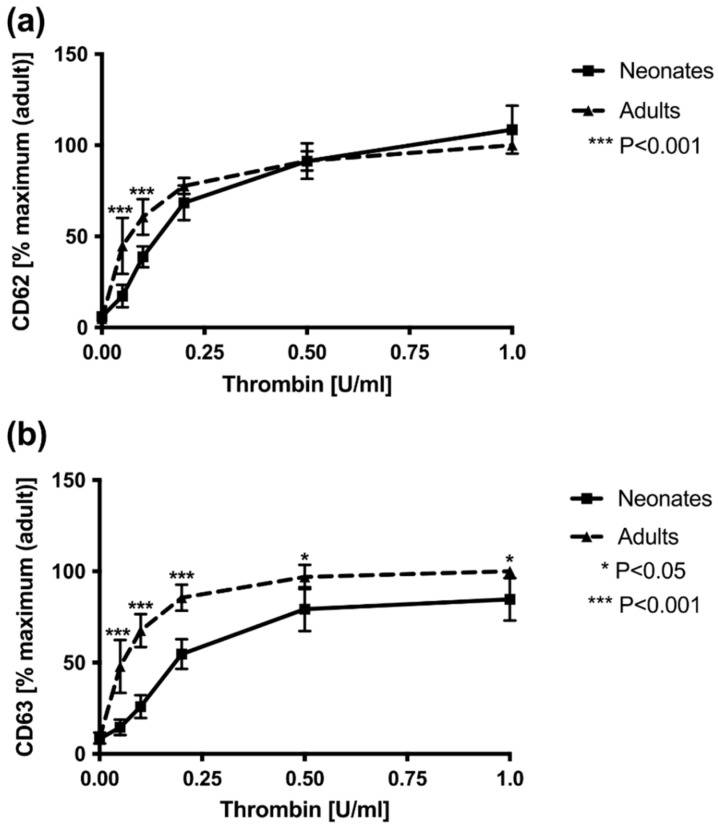
Surface expression of CD62 (**a**) andCD63 (**b**) in diluted platelet-rich plasma obtained from adult and neonatal samples ex vivo and after stimulation with increasing concentrations of thrombin. Results are presented as mean ± SD. Data (*n* = 11 per cohort) are normalized to maximal response measured in adult control samples run in parallel. *** *p* < 0.001; * *p* < 0.05.

Flow cytometric analysis of CD62 and CD63 expression showed differences in neonatal platelets compared to adult platelets after stimulation with low concentrations of thrombin (Figure 1). CD63 expression was lower in neonatal platelets compared to adult platelets at all employed thrombin concentrations with highly significant differences at low thrombin concentrations and persistently reduced CD63 expression after full stimulation with thrombin (Table S1, Figure 1b).

A lower CD62 expression on neonatal platelets compared to adult platelets was found with 0.05 U/mL thrombin (adult: 44.8 ± 19.4%; neonatal: 17.3 ± 6.2%; *p* < 0.001) and 0.1 U/mL thrombin (adult: 60.7 ± 9.7%; neonatal: 38.8 ± 5.8%; *p* < 0.001). However, no difference was found in CD62 expression after stimulation with the highest concentration of thrombin (1 U/mL), indicating comparable secretion of after exhaustive activation (Figure 1a).

### 3.2. G-Protein Expression of Neonatal and Adult Platelets

**Figure 2:**
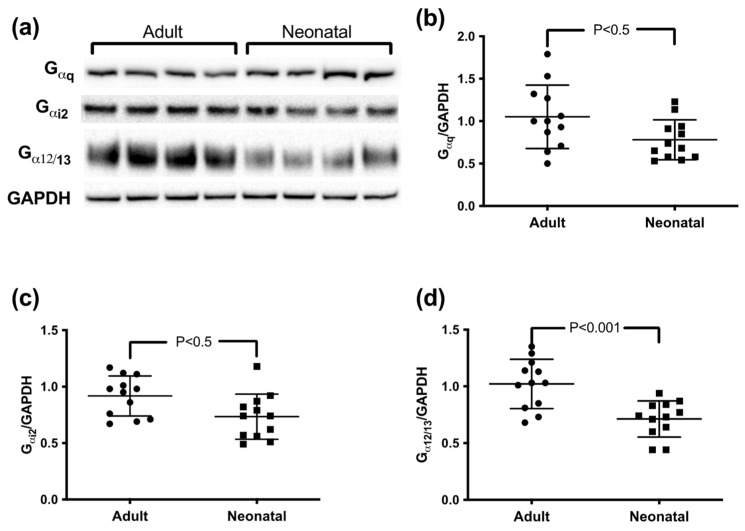
Expression of G-proteins in adult and neonatal platelets. (**a**) Representative blot lanes from Western blot analyses of G_αq_, G_αi2_, and G_α12/13_ as well as GAPDH as loading and staining control. (**b**–**d**) Statistical depiction of all G-protein blot densities normalized to GAPDH. Bars show mean ± SD. Statistical differences were determined with Student’s *t*-test.

Densitometric Western blot analysis revealed differential G-protein expression in adult and neonatal platelets depending on the G-protein isoform (Figure 2a). The average of G_αq_ expression normalized to GAPDH was only slightly lower on neonatal platelets compared to adult platelets (adult: 1.05 ± 0.38 AU; neonatal: 0.78 ± 0.24 AU; *p* < 0.05) (Figure 2b). Similarly, analysis of G_αi2_ showed only minor differences between adult and neonatal expression levels (adult: 0.92 ± 0.18 AU; neonatal: 0.73 ± 0.20 AU; *p* < 0.05) (Figure 2c). The most pronounced difference was observed in G_α12/13_ expression with significantly lower levels in neonatal platelets (adult: 1.022 ± 0.21 AU; neonatal: 0.71 ± 0.16 AU; *p* < 0.001) (Figure 2d).

### 3.3. Rho Activation and Correlations

**Figure 3:**
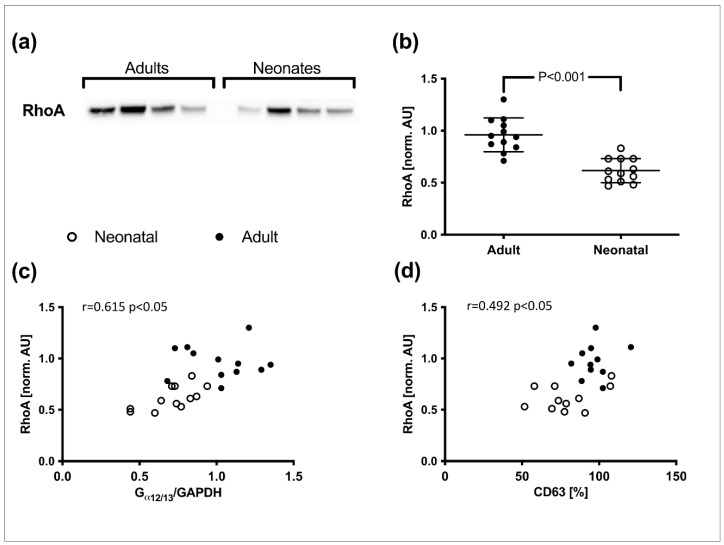
Rho activation in adult and neonatal platelets after stimulation with thrombin (0.5 U/mL). (**a**) Representative blot lanes from Western blot analyses after Rho pull-down from thrombin stimulated adult and neonatal platelets. (**b**) Statistical depiction of activated Rho blot-band densities normalized to the average of adult values. Bars show mean ± SD. Statistical difference was determined with Student’s *t*-test. (**c**) Scatter plot showing positive correlation between Rho activation and G_α12/13_ expression. (**d**) Scatter plot showing positive correlation between Rho activation and CD63 expression after thrombin stimulation (0.5 U/mL).

Submaximal stimulation with thrombin (0.5 U/mL) resulted in significantly lower activation of Rho in neonatal platelets compared to adult platelets (adult: 0.96 ± 0.16 AU; neonatal: 0.62 ± 0.12 AU; *p* < 0.001) (Figure 3a,b). Pearson analysis revealed a positive correlation between normalized G_α12/13_ expression levels and Rho activation (r = 0.615, *p* < 0.05) (Figure 3c). A slight correlation was observed between CD63 expression after stimulation with 0.5 U/mL thrombin and Rho activation (r = 0.4923, *p* < 0.05) (Figure 3d). No correlation was found between G_α12/13_ expression levels (r = 0.345, *p* = 0.1156) and CD 63 expression.

## 4. Discussion

Neonatal platelet hypofunction has been observed in multiple studies. Interestingly, the underlying impairment varies depending on the type of agonist. Equivalently, neonatal secretion defects may be a side effect of impaired platelet activation by a specific agonist or a general phenomenon caused by reduced granule content or an immature vesicle fusion machinery.

Platelet secretion is a major contributor to overall platelet function, since the shedding of additional platelet agonists and thrombotic facilitators (e.g., adenine nucleotides, serotonin, and PolyP) are required for fully functional primary and secondary hemostasis. Therefore, it is conclusive that secretion defects contribute to neonatal platelet hypoaggregability.

Consistent with other groups, we found impaired secretion of dense granules marked by reduced expression of CD63 after activation of neonatal platelets [3,35]. Another study focusing on electron microscopy analysis of neonatal platelets revealed a reduced number of dense granules that may contribute to the observed impairment [36]. It has been proposed that this secretion defect is secondary to platelet activation due to birth stress that causes partial platelet degranulation. However, no signs of partial activation of neonatal platelets during delivery have been observed [37,38].

We analyzed G-protein subtypes known to be coupled to thrombin receptors and found only minor differences in G_q_ and G_i_ expression between adult and neonatal platelets, but significantly lower G_12/13_ expression in the latter. Since G_12/13_ is known to regulate dense granule release via RhoA [32], we analyzed Rho activation of neonatal and adult platelets after thrombin stimulation and observed a marked reduction in neonatal platelets. Interestingly, we found a correlation between Rho activation and respective G_12/13_ expression. This is counterintuitive because G_q_ can also activate Rho, obfuscating a direct relationship between G_12/13_ and Rho under normal circumstances. However, in the case of neonatal platelets, the previously reported lack of GTPase activity in the G_αq_-subunit [26] may shift the predominance of Rho activation to G_12/13_, explaining the significant correlation.

We did not observe a direct correlation between respective expression of G_12/13_ and CD63 expression, which is influenced by multiple factors. However, the reduced G_12/13_ expression coincided with reduced CD63 expression at the same thrombin concentration. Given the known contribution of G_12/13_ and RhoA to dense granule secretion [32], we conclude that lower G_12/13_ expression is a specific impairment of neonatal platelets that may contribute to reduced dense granule release secondary to thrombin exposure. This impairment may also extend to TXA_2_-receptors and other GPCRs coupled to G_12/13_, which is a subject for further investigation.

A limitation of this study is the use of cord blood platelets as a surrogate for neonatal peripheral blood. Drawing the amount of venous blood required for the employed assays from healthy neonates was not ethically justifiable. Differences in the severity of the secretion defect compared to data from venous blood by Herken et al. may be attributable to methodological variations. While Herken et al. stimulated only PAR1 with a synthetic thrombin receptor activating peptide (TRAP) [3], we employed thrombin to achieve dual receptor activation (PAR1 and PAR4).

We acknowledge that the expression of some platelet-activating or -inhibiting factors in cord blood may change rapidly after birth, resulting in altered platelet function. We have shown that spiking prostaglandin E2 levels before birth add an inhibitory stimulus on platelets that rapidly declines due to catabolism via pulmonary circulation [39]. However, in this study we used washed platelet suspension to study G_12/13_ expression and thrombin-induced Rho activation. Therefore, we can exclude differences in plasmatic factors in cord blood and neonatal peripheral blood that influence the observed impairment of the G_12/13_ pathway.

## 5. Conclusions

We observed lower expression of G_12/13_ in neonatal platelets correlating with reduced Rho activation. This impairment of the G_12/13_ signaling pathway coincided with reduced dense granule secretion after thrombin stimulation, which argues for a contribution to the observed secretion defects of neonatal platelets. Taking the fragility of neonatal coagulation into account, attenuated platelet secretion may not be a mere sign of immaturity but an adaption to sustain the fragile hemostatic balance after birth.

## Data Availability

The data presented in this study are available on reasonable request from the corresponding author. Reagents and detailed methods of all procedures are provided in Section 2 of this manuscript or cited accordingly.

## Supplementary Materials

The following are available online at https://www.mdpi.com/article/10.3390/cells11162563/s1, Table S1: CD63 expression in adult and neonatal platelets after stimulation with increasing amounts of thrombin.
