# Supplementary material for: Neonatal Platelets: Lower G12/13 Expression Contributes to Reduced Secretion of Dense Granules
Source: Cells. 2022 Aug 17;11(16):2563. doi: 10.3390/cells11162563 (PMC9406762; doi:10.3390/cells11162563)
Supplement: Supplementary file 1 [file cells-11-02563-s001.zip › cells-1851855-supplementary.pdf]

Supplementary material

Table S1. CD63 expression in adult and neonatal platelets after stimulation with increasing amounts of thrombin. Results are presented as mean±sd. Data (n= 11 per cohort) are normalized to maximal response measured in adult control samples run in parallel.

Thrombin concentrations [U/ml]	Adult [%]	Neonatal [%]	P
0	8.74±3.01	8.26±2.41	>0.999
0.05	47.9±14.5	14.6±4.3	<0.001
0.1	67.5±9.0	25.9±6.3	<0.001
0.2	85.6±7.2	54.7±8.1	<0.001
0.5	96.9±6.71	79.4±12.1	0.0102
1	100*	84.7±11.6	0.0340

*normalized percentage
